# Strongly coupled spins of silicon-vacancy centers inside a nanodiamond with sub-megahertz linewidth

**DOI:** 10.1515/nanoph-2023-0927

**Published:** 2024-03-13

**Authors:** Marco Klotz, Richard Waltrich, Niklas Lettner, Viatcheslav N. Agafonov, Alexander Kubanek

**Affiliations:** Institute for Quantum Optics, 9189 Ulm University, 89081 Ulm, Germany; GREMAN, UMR 7347 CNRS, INSA-CVL, Tours University, 37200 Tours, France

**Keywords:** quantum optics, nanodiamond, silicon-vacancy center, electron-spin, nuclear-spin

## Abstract

The search for long-lived quantum memories, which can be efficiently interfaced with flying qubits, is longstanding. One possible solution is to use the electron spin of a color center in diamond to mediate interaction between a long-lived nuclear spin and a photon. Realizing this in a nanodiamond furthermore facilitates the integration into photonic devices and enables the realization of hybrid quantum systems with access to quantum memories. Here, we investigated the spin environment of negatively charged silicon-vacancy centers in a nanodiamond and demonstrate strong coupling of its electron spin, while the electron spin’s decoherence rate remained below 1 MHz. We furthermore demonstrate multi-spin coupling with the potential to establish registers of quantum memories in nanodiamonds.

## 1 Introduction

In the future, quantum-based networks can provide secure communication or distributed quantum computing [1], [2], [3], [4]. One of the remaining challenges is finding a scalable network node, which can process, distribute, and store quantum information, efficiently. Qubits based on solid-state quantum emitters offer advantages in terms of scalability. First small networks, for example, based on negatively charged nitrogen-vacancy centers in diamond (NV^−^) have been realized in a pioneering work [5]. However, the NV^−^ is prone to perturbations from external fields and the rate of coherent photons is low [6], [7]. In contrast, group-IV defects like the negatively charged silicon-vacancy center (SiV^−^) are insensitive to external electric fields and show intrinsically identical emitter [8], [9], [10]. Recent results demonstrated coherent control of the SiV^−^ spin with coherence times in the ms range when operating at mK temperatures [11]. Increasing the operation temperature is desirable to reduce technical overhead. One potential solution is SiV^−^ in nanodiamonds (NDs) with modified electron–phonon interactions [12], which can be integrated into hybrid quantum systems such as photonic crystal cavities [13], [14] or plasmonic waveguides [15]. In this letter, we show the observation of spins in a ND strongly coupled the electron spin of a SiV^−^ center. The coupling strength of one of the spins is in good agreement to theoretical modeling of a nearest neighbor ^13^C nuclear spin [16]. We further show that for a SiV^−^ in a ND the main mechanism for decoherence of its spin qubit, which is phonon-mediated dephasing, can already be mitigated at temperatures of around 4 K. The resulting suppressed decoherence rate, access to a local memory, and fast initialization rates lay the foundations for coherent control of an integratable hybrid quantum network node based on SiV^−^ s in NDs.

## 2 Results

**Figure 1:**
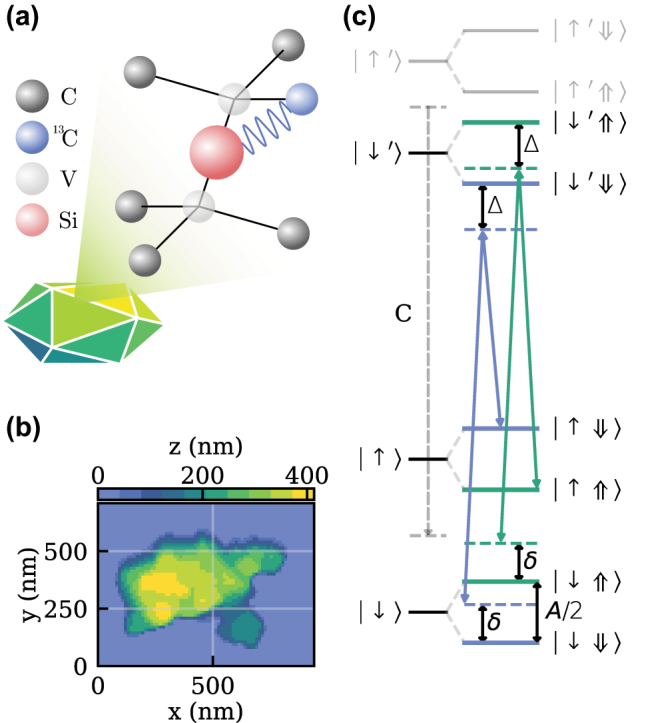
SiV center in a ND and partial electronic structure of the SiV. (a) Illustration of a SiV^−^ with a coupled ^13^C nuclear spin inside a ND. The implied shear represents the presence of strain in the host crystal. (b) AFM scan of the nanodiamond agglomeration containing the SiV^−^s under consideration. (c) Partial level scheme for transition C of the SiV^−^. The four levels originating from the Zeeman effect are labeled as |↓⟩, |↑⟩, |↓′⟩, and |↑′⟩. The corresponding hyperfine levels are indicated with an additional ⇓ or ⇑. Λ systems connecting the correspondingly involved electron and nuclear spins are differently colored. Single- and two-photon detunings are labeled with Δ and *δ*, respectively. Other transitions arising from A, B, and D are not shown for simplicity.

The SiV^−^ is a point defect in the diamond lattice, where a silicon atom (Si) with an excess electron is situated between two adjacent carbon vacancies (V) as schematically depicted in Figure 1(a). An atomic force microscope scan of the ND containing the investigated SiV^−^ revealed an agglomeration of NDs as shown in Figure 1(b). We estimate the size range of individual NDs to be between 50 nm and 250 nm. The agglomeration is small enough to be integrated into a cavity system [17]. For individual NDs within the agglomeration, a modified phonon density of states (PDOS) at low strain is possible [12]. The electronic level scheme of the SiV^−^ consists of four spin-degenerate orbital states, two of which form the ground-state (GS) and excited-state (ES), respectively. As a consequence, four optically active transitions arise, which we label as A, B, C, and D. For the remainder of the text, we only use transition C, for which the spin levels are depicted in Figure 1(c). The spin degeneracy of the GS and ES levels can be lifted by applying a magnetic field, giving access to an electron spin qubit, e.g., the one labeled by |↓⟩ and |↑⟩ [18], [19]. When using such a spin-qubit at liquid helium temperature, its coherence time is mainly limited through phonon-induced dephasing. The latter can be mitigated by either cooling the system to mK temperatures [11], changing the PDOS [12], or increasing the GS splitting, which suppresses phonon absorption [20], [21]. The use of NDs is an appealing choice as a host for the SiV^−^, since they can combine two of the mentioned effects to increase spin-coherence times at temperatures around 4 K. The reduced size can modify the PDOS and commonly present strain in NDs results in an increased GS splitting. We, therefore, investigate spectrally shifted SiV^−^ where high strain can be expected [20] and studied them for their optical and spin-coherence properties.

**Figure 2:**
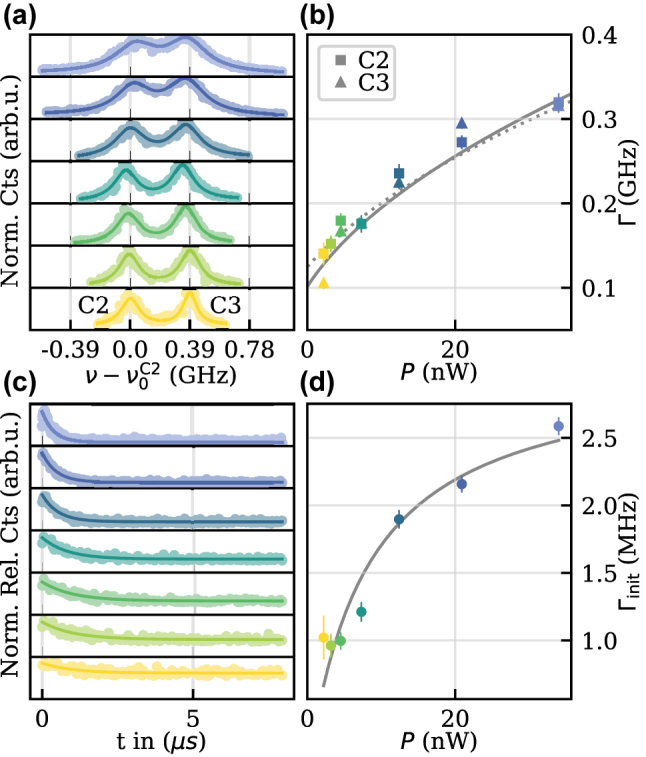
Optical properties of a SiV-center. (a) The data points show power-dependent PLE scans of the spin-preserving transitions C2 and C3 with the frequency relative to $\nu_{0}^{C_{2}}$ of transition C2. The solid lines are double-Lorentzian fits to the data and reveal a splitting of 390 ± 4 MHz for the lowest excitation power *P*. (b) Fitted linewidth as a function of *P* with squares and triangles representing C2 and C3, respectively. The dotted and solid lines are the respective square-root fit. (c) Normalized fluorescence during spin initialization for increasing excitation powers with the solid lines being an exponential fit. For the highest power (lower panel), the initialization time is 387 ± 10 ns with a fidelity of 0.806 ± 0.004. (d) The extracted initialization rates Γ_init_ as a function of *P*.

The NDs were coated onto a sapphire substrate with good thermal conductivity. The sample was then cooled to liquid helium temperatures in a continuous flow-cryostat and investigated using a home-built confocal microscope. Four permanent magnets in a Hallbach-configuration designed for an in-plane field strength of around 400 mT were used to lift the spin degeneracy. Individual transitions of the SiV^−^ were addressed by photo-luminescence excitation spectroscopy (PLE). After finding a suitable SiV^−^, the optical linewidths of the two spin-preserving transitions, labeled as C2 and C3, were investigated by PLE with varying power (*P*), as shown in Figure 2(a). The frequency splitting between C2 and C3 390 ± 4 MHz was determined by a double-Lorentzian fit. Figure 2(b) shows the fitted power-dependent linewidth Γ of C2 and C3. We extrapolated the linewidth to zero power using $\Gamma \left(P\right)=\Gamma_{0}\sqrt{s+1}$ , where Γ_0_ is the linewidth at zero power and *s* = *P*/*P*_sat_ with the saturation power *P*_sat_. The fit resulted in $\Gamma_{0}^{\text{C}2}=125\;\pm \;9\;\text{MHz}$ and $\Gamma_{0}^{\text{C}3}=101\;\pm \;18\;\text{MHz}$ , respectively. The saturation powers turned out as $P_{\text{sat}}^{\text{C}2}=5.8\;\pm \;1.2\;\text{nW}$ and $P_{\text{sat}}^{\text{C}3}=3.4\;\pm \;1.6\;\text{nW}$ , respectively. Firstly, Γ_0_ is close to Fourier limits for commonly found lifetimes inside NDs, suggesting excellent optical quality. Secondly, the narrow linewidths compared to 390 MHz allow for a strong drive without significant cross-talk between C2 and C3.

We proceeded by measuring the initialization rate of the spin for each excitation power. To this end, we pumped the population out of a thermal maximally mixed equilibrium state with a resonant pulse of varying power. The resulting fluorescence, shown in Figure 2(c), was then fitted with an exponential decay. From the fit, we evaluated the initialization fidelity by comparing the maximum fluorescence at the beginning of the pulse with the fluorescence at the end of the pulse, corresponding to the steady state population under resonant drive. In addition, the corresponding initialization rates Γ_init_ are shown in Figure 2(d). For the highest power, we measured an initialization time of 387 ± 10 ns and a fidelity of 0.806 ± 0.004. From the power dependence of Γ_init_, we determined the spin-branching ratio *η* = 22.7 ± 1.2 by fitting $\Gamma_{\text{init}}=\frac{1}{\eta }\frac{s}{\left(1+s\right)}\frac{\Gamma_{0}^{\text{C}2}}{2}$ , where $\Gamma_{0}^{\text{C}2}$ and $P_{\text{sat}}^{\text{C}2}$ are taken from the previously fitted PLE measurements [22].

Extending the measurement procedure of Figure 2(c) to multiple consecutive resonant pulses with increasing temporal spacing probes the exponentially recovering spin population *T*_1_. Fitting an exponential to the peak intensities in each fluorescence pulse resulted in *T*_1_ = 2.0 ± 0.7* *μs, which ultimately limits spin coherence. In addition, *T*_1_ gives insight into the orientation of the SiV^−^ symmetry axis with respect to the magnetic field, where a misalignment leads to spin mixing and thus shorter relaxation times [23]. From the relatively short *T*_1_ together with the spin-branching *η*, we estimate only a moderate alignment. A tunable magnet system or nanomanipulation [24] may improve the alignment and thus increase the maximum achievable coherence time and initialization fidelity in future experiments [23].

**Figure 3:**
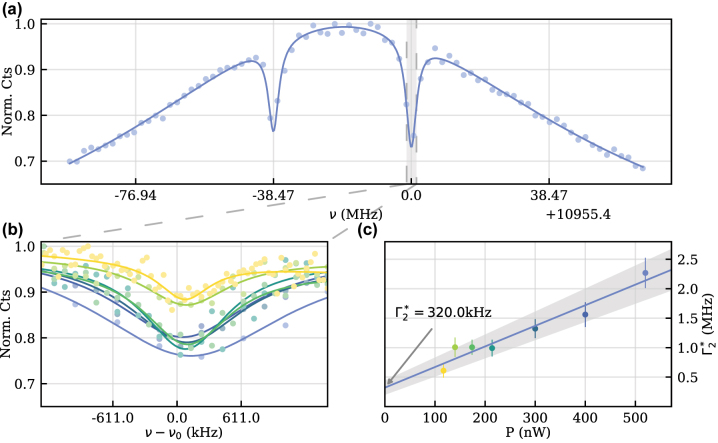
Coupling and coherence properties of a SiV-center. (a) CPT measurement of a SiV^−^-center showing two dips, indicating a nearby ^13^C nuclear spin. The solid line is a triple-Lorentzian fit to the data points. The resulting Zeeman splitting is around 11 GHz and the splitting between the two dips is *A* = 38.47 ± 0.12 MHz. (b) Measurement of the CPT width of the dip highlighted by the gray area in (a) for varying power. (c) Power-dependent CPT width $\Gamma_{2}^{*}$ . A linear fit yields a rate of $\Gamma_{2}^{*}=320\;\pm \;120\;\;\text{kHz}$ at zero power.

To probe the spin-coherence, we used coherent population trapping (CPT). Here, the laser resonantly (Δ = 0, see Figure 1(c)) drove a spin-flipping transition. Simultaneously, an electro-optical modulator (EOM) generated sidebands from which one was swept over the corresponding spin-preserving transition. If the Raman condition (*δ* = 0) is fulfilled, the system is pumped into a dark state quenching the fluorescence signal. For the studied SiV^−^, two dips with a frequency splitting of *A* = 38.47 ± 0.12 MHz were present, as shown in Figure 3(a). The splitting A is composed of two terms, the parallel coupling term *A*_‖_ and the perpendicular coupling term *A*_⊥_. The observed splitting is in close agreement with a theoretically predicted strongly hyperfine-coupled next-nearest neighbor ^13^C nuclear spin with a coupling strength of *A*_‖_ = 37 MHz [16].

Furthermore, since the dip’s linewidth gives insight on the spin’s dephasing rate, we performed a power-dependent measurement, which is shown in Figure 3(b). Fitting the data with a Lorentzian and extracting the linewidth $\Gamma_{2}^{*}$ allowed us to linearly extrapolate the dephasing rate to zero laser power to suppress power-induced broadening. As a result, we obtained a zero-power linewidth of $\Gamma_{2}^{*}=320\;\pm \;120\;\;\text{kHz}$ . This is approximately a ten-fold reduced decoherence rate compared to previously reported measurements in bulk diamond at similar temperatures with $\Gamma_{2}^{*}\approx 4.5\;\text{MHz}$ [25], [26], [27], and a minor improvement to measurements in a strain-engineered nano-beam, which reported $\Gamma_{2}^{*}=640\;\text{kHz}$ [20], [21]. Because the minimal size of individual NDs is 50 nm, we do not expect a significantly changed PDOS in a high strain environment. Instead, we attribute the improved coherence time mainly to the suppressed phonon absorption caused by the increased GS splitting. Combining the latter improvements of spin coherence in high strain environments with the present spin transition frequency of roughly 11 GHz and the spin-cycling transitions’ splitting of 390 MHz suggests a comparably highly strained SiV^−^-center with a GS splitting on the order of 500 GHz [20].

**Figure 4:**
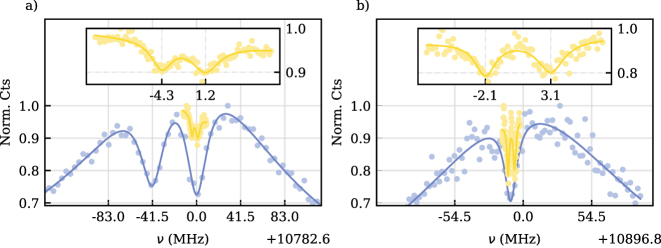
CPT measurements of two more SiV^−^ with low and high optical power, indicated in yellow and blue, respectively. (a) Shows two dips, split by *A* = 41.5 ± 0.7 MHz. Low-power measurements reveal a coupled system with an additional splitting of *A*′ = 5.47 ± 0.17 MHz. (b) Measurements show a double dip, split by 5.20 ± 0.14 MHz, indicating a weakly coupled C13 nuclear spin.

We further investigated the spin environment in the same agglomeration of NDs by performing CPT measurements on different SiV^−^-centers. The measurement revealed coupling of multiple spins, as shown in Figure 4. For example, the SiV^−^ in Figure 4(a) displayed two dips split by *A* = 41.5 ± 0.7 MHz. Measurements with reduced power resolved two more dips, split by *A*′ = 5.47 ± 0.17 MHz as shown in the inset. They exhibited linewidths of 3.2 ± 0.4 MHz and 4.3 ± 0.5 MHz, for the left and right dip, respectively. The distinct linewidths suggest coupling to two other surrounding SiV^−^-centers’ electron spins with coherence properties commonly found with SiV^−^ in low to moderate strain bulk diamond [25], [26], [27]. Assuming a dipolar electron–electron coupling would correspond to a distance on the order of 10 nm, reasonable for the size of the ND and density of SiV^−^-centers under study. Another SiV^−^, displayed in Figure 4(b), exhibited two individual dips at low optical power with linewidths of 2.0 ± 0.3 MHz and 2.6 ± 0.5 MHz, for the left and right, respectively. In contrast to the previous SiV^−^s’ multi-dip structure with distinct linewidths, the fact that the present SiV^−^ has closely matching and relatively narrow linewidths is indicative of a coupled nearby ^13^C nuclear spin with a coupling strength of 5.20 ± 0.14 MHz [21]. In this case, the distance between the two coupled spins is on the order of 1 Å [21].

## 3 Conclusions

The presented results open up new possibilities to utilize the electron and nuclear spin environment of SiV^−^-centers in NDs as an elementary unit of diamond-based qubits with integrability into photonic platforms, like photonic crystal or open microcavities [14], [17], [28], [29]. Depending on the size of the ND, evanescent coupling to other systems such as plasmonic waveguides is also possible, where high Purcell factors can be expected [15]. In addition, phononic [30] or optomechanical [31] platforms could be used to further reduce phonon-induced dephasing via a modified PDOS while maintaining or enhancing the photon collection efficiency. The thereby formed hybrid quantum system enables efficient mapping of the quantum state of the electron spin to flying photonic qubits as well as coupling to local memory units consisting of nearby nuclear spins. Our work also suggests that the access to small spin registers is feasible for SiV^−^-centers in NDs.

Increased strain, e.g., caused by hydrostatic pressure might further improve coherence times [32]. Relying on intrinsic, nonuniform natural strain challenges the scalability of spectrally matched emitters. However, either postselection of NDs, strain tuning [20], or electric field-induced fine-tuning of the emission wavelength [33] relaxes spectral constraints. Our results are also applicable to other group IV defects in diamond with intrinsically higher GS splitting, such as the germanium vacancy (GeV) and tin vacancy (SnV). For example, highly strained GeV centers in NDs with a GS splitting of 870 GHz have been reported in Ref. [34]. The dephasing rates bring coherent control of different spin qubits in NDs, either by means of direct microwave drive [35] or all-optical control using a Raman-type lambda scheme [22], into reach. Additionally, temperatures around 4 K within a flow-cryostat become sufficient. For the system parameters in our measurements, an all-optical Rabi driving strength in the order of several MHz can be expected, comparable to driving rates achieved with a microwave [35]. Additionally, the high sensitivity enables to detect close-by electron or nuclear spins. Furthermore, with coherent electron spin control, direct [36] or indirect [37] control of a nuclear spin becomes possible. Mapping information of the electron spin to a strongly coupled long-lived nuclear spin establishes SiV^−^-centers in NDs as a viable candidate for an interchangeable hybrid quantum memory.
